# Knock-out of *SO1377 *gene, which encodes the member of a conserved hypothetical bacterial protein family COG2268, results in alteration of iron metabolism, increased spontaneous mutation and hydrogen peroxide sensitivity in *Shewanella oneidensis *MR-1

**DOI:** 10.1186/1471-2164-7-76

**Published:** 2006-04-06

**Authors:** Weimin Gao, Yongqing Liu, Carol S Giometti, Sandra L Tollaksen, Tripti Khare, Liyou Wu, Dawn M Klingeman, Matthew W Fields, Jizhong Zhou

**Affiliations:** 1 Environmental Sciences Division, Oak Ridge National Laboratory, Oak Ridge, TN 37831, USA; 2 Biosciences Division, Argonne National Laboratory, Argonne, IL 60439, USA; 3 Department of Microbiology, Miami University, Oxford, OH 45056, USA; 4 Institute for Environmental Genomics and Department of Botany and Microbiology, University of Oklahoma, Norman, OK 73019, USA

## Abstract

**Background:**

*Shewanella oneidensis *MR-1 is a facultative, gram-negative bacterium capable of coupling the oxidation of organic carbon to a wide range of electron acceptors such as oxygen, nitrate and metals, and has potential for bioremediation of heavy metal contaminated sites. The complete 5-Mb genome of *S. oneidensis *MR-1 was sequenced and standard sequence-comparison methods revealed approximately 42% of the MR-1 genome encodes proteins of unknown function. Defining the functions of hypothetical proteins is a great challenge and may need a systems approach. In this study, by using integrated approaches including whole genomic microarray and proteomics, we examined knockout effects of the gene encoding SO1377 (gi24372955), a member of the conserved, hypothetical, bacterial protein family COG2268 (Clusters of Orthologous Group) in bacterium *Shewanella oneidensis *MR-1, under various physiological conditions.

**Results:**

Compared with the wild-type strain, growth assays showed that the deletion mutant had a decreased growth rate when cultured aerobically, but not affected under anaerobic conditions. Whole-genome expression (RNA and protein) profiles revealed numerous gene and protein expression changes relative to the wild-type control, including some involved in iron metabolism, oxidative damage protection and respiratory electron transfer, e. g. complex IV of the respiration chain. Although total intracellular iron levels remained unchanged, whole-cell electron paramagnetic resonance (EPR) demonstrated that the level of free iron in mutant cells was 3 times less than that of the wild-type strain. Siderophore excretion in the mutant also decreased in iron-depleted medium. The mutant was more sensitive to hydrogen peroxide and gave rise to 100 times more colonies resistant to gentamicin or kanamycin.

**Conclusion:**

Our results showed that the knock-out of *SO1377 *gene had pleiotropic effects and suggested that SO1377 may play a role in iron homeostasis and oxidative damage protection in *S. oneidensis *MR-1.

## Background

*Shewanella oneidensis *MR-1 is a facultative, gram-negative bacterium capable of coupling the oxidation of organic carbon to a wide range of electron acceptors such as oxygen, nitrate and metals, and has potential for bioremediation of heavy metal contaminated sites [1-5]. The complete 5-Mb genome of *S. oneidensis *MR-1 was sequenced by The Institute for Genomic Research (TIGR), and standard sequence-comparison methods revealed that approximately 42% of the MR-1 genome encodes proteins of unknown function [6]. Whole-genome sequence analyses from a variety of other microorganisms also indicate that 30–60% of the identified genes encode proteins of unknown function. Defining the functions of hypothetical proteins is a great challenge and may need a systems approach. A structure-based approach provides clues about the biochemical functions of a hypothetical protein [7,8]. The emergent whole genomic microarray and proteomics are providing new experimental tools for our understanding of functions and regulations of novel proteins in a context of global response to environmental stresses and genetic disturbance [9,10]. The patterns of gene expression changes are giving clues into the mechanisms of action of response. Combining with traditional approaches, the functions of novel proteins could be elucidated more efficiently by an integrated methodology.

We initiated a study to characterize the functions of a hypothetical protein of *S. oneidensis *MR-1. The protein, which is designated SO1377 (gi24372955), was initially identified during a 2-D gel expression study of a *Shewanella *FUR (ferric uptake regulator) mutant. During aerobic respiration of this FUR mutant, results showed the expression of SO1377 was down-regulated [11]. SO1377 contains 592 amino acids, and homologues are apparent in other bacteria, such as YqiK (gi3915528) of *Escherichia coli *and a putative exported protein of *Salmonella enterica*, with 45% and 44% sequence identity, respectively. PHI/PSI-BLAST search results showed that SO1377 is included in a conserved, hypothetical, bacterial protein family COG2268. Similarity search also showed that the N-terminal sequence of SO1377 belongs to the PHB (prohibitin homologues) family and the SPFH (Stomatin, Prohibitin, Flottlin and HflKC) domain /Band 7 superfamily. Prohibitin (PHB) are ubiquitously expressed and highly conserved proteins with homologues found in organisms ranging from yeast to humans. In the yeast *Saccharomyces cerevisiae*, it was suggested that the PHB proteins were involved in mitochondrial respiratory complex assembly and played a role in the biogenesis of newly synthesized subunits of mitochondrial respiratory enzymes [12-14]. HflC and HflK of *E. coli *are two members of the SPFH domain/Band 7 superfamily. They associate with and negatively regulate the FtsH AAA protease and participate in the lysogenic decision during bacteriophage λ infection [15]. Other members of SPFH domain/Band 7 superfamily also include: stomatin, one of the major integral membrane proteins of human erythrocytes [16], and the caveolae-associated flotillins [17]. Although proteins of both stomatin and flotillin families appear to be involved in important biological processes, very little is known about their *in vivo *functions.

According to the sequence annotation [6], genes encoding SO1377 and SO1376 might be in the same operon. The *SO1377 *and *SO1376 *genes are only separated by 23-bp. On the downstream of the SO1377-encoding gene, a ρ-independent transcriptional terminator is identified. Thus, *SO1377 *and *SO1376 *might be on the same transcriptional unit. The genes encoding SO1376 and 1377 are flanked by *SO1378 *and *SO1375 *loci. SO1376 is annotated as a putative oxidoreductase of 212 amino acids. A PHI/PSI-BLAST search showed that SO1376 belongs to DUF1449, a protein family consisting of several bacterial proteins of around 210 residues in length. The function of DUF1449 family is unknown. Previously, a computational analysis of secondary and 3-D structure of SO1377 was carried out and revealed that this protein had potential functions in the formation of protein complexes at the inner bacterial cell membrane, ATP/GTP binding, nucleotide binding, protein transport and molecular chaperone [18].

In this study, we continued our efforts to elucidate the functions of SO1377 by generating an in-frame deletion mutant of this gene, followed by a series of experiments to characterize its phenotypic and physiological changes compared with the wild type strain. Our results showed that the knock-out of *SO1377 *gene had pleiotropic effects and suggested that SO1377 perhaps plays a role in the iron homeostasis and oxidative damage protection in *S. oneidensis *MR-1.

## Results

### Generation of in-frame deletion mutant of *SO1377 *gene

**Figure 1:**
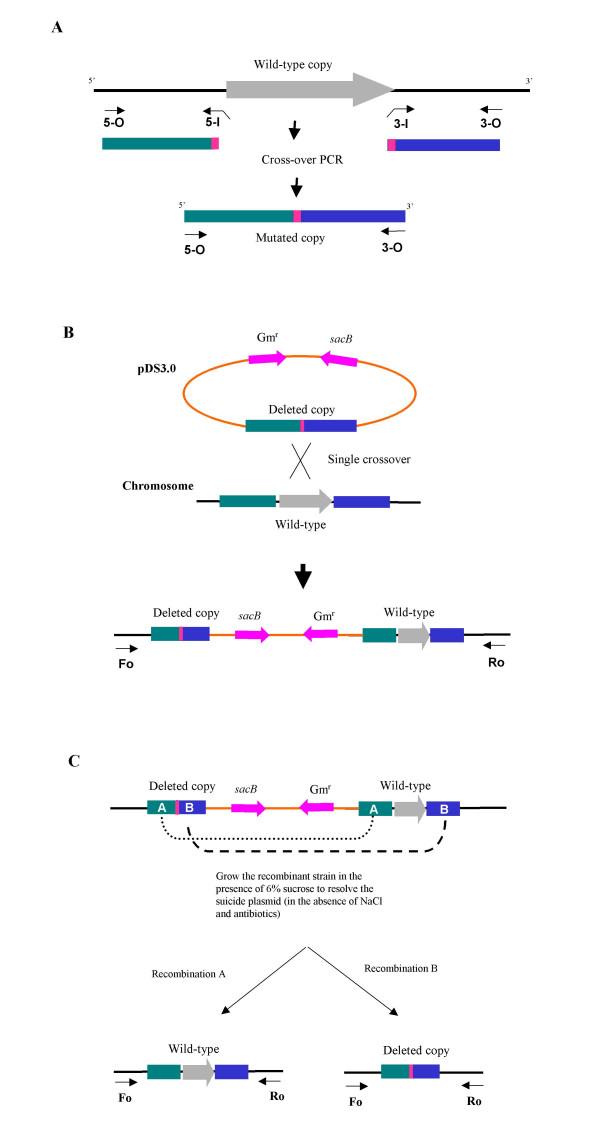
Schematic presentation of generation of in-frame deletion mutant of *SO1377 *gene A. Generation of a deletion construct using asymmetric crossover PCR; B. Integration of the suicide plasmid during a single crossover event; C. Generation of in-frame deletion during the excision of the suicide plasmid. More detail about this construction process was described under "methods".

The suicide vector, pDS3.0, was used in generation of deletion mutant of *SO1377 *gene (Table 1). pDS3.0 was derived from the suicide plasmid pCVD442 by a blunt ligation of *Sal*I digested pCVD442 with a gel purified 1597 bp fragment of *Mlu*I digested pBSL142, in which harbors the gentamicin resistance gene. By crossover PCR, a 1.1 kb DNA fragment containing the mutated *SO1377 *gene, with a 1496-bp deletion, was constructed (Fig. 1A). This deletion construct was ligated with pDS3.0 and then transferred into *E. coli *S17-1/λ_pir _by electroporation. By employment of colony PCR, the resultant plasmid was confirmed and designated as pWG3 and the *E. coli *host strain WG1 (Table 1). By transconjugation between donor strain *E. coli *WG1 and recipient strain *S. oneidensis *DSP-10, the constructed suicide plasmid pWG3 containing the deletion mutation of *SO1377 *gene was transferred and integrated into the chromosome of DSP-10 (Fig. 1B). The occurrence rate of recombinants generated from transconjugation/integration of plasmid pWG3 was 4.5 × 10^-8^. By growing the recombinant strain WG2 on LB agar in the presence of 6% sucrose, a total of 54 colonies appeared after two days incubation at 30°C. Of these colonies, 33 lost gentamicin resistance, indicating they had resolved the suicide plasmid pWG3 from the chromosome of WG2 (Fig. 1C). Further colony PCR revealed about 50% of 33 colonies had the deletion copy of *SO1377 *gene in place of the wild-type copy. Since initial growth assays showed that all deletion mutants had the similar growth deficiency compared with that of the wild-type control, in follow-up experiments we focused on one deletion mutant, WG3, for further characterization of phenotypes by using whole genomic microarray and proteomics technologies.

### Growth features of the deletion mutant

**Figure 2:**
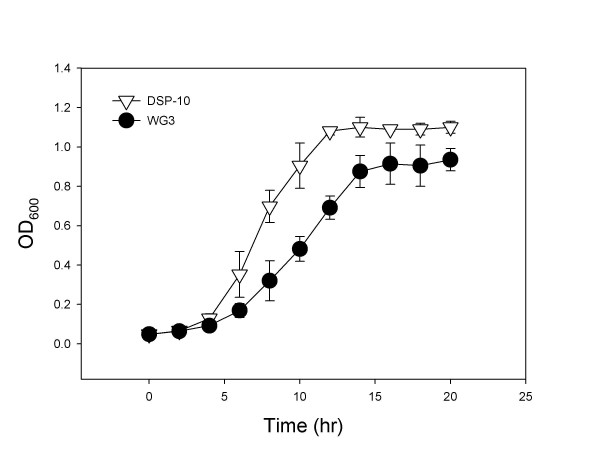
Growth curve of both *SO1377 *wild-type strain DSP-10 and deletion mutant WG3 in medium MR2A under aerobic conditions at 30°C. Note that the maximum growth peak reached by the wild-type control is around 1.2 while the mutant is 1.0.

When grown under aerobic conditions in MR2A medium, the deletion mutant WG3 had a growth deficiency compared to the parental strain DSP-10 (Figure 2). Under this condition, the average generation time of WG3 was 2.9 hours while that of DSP-10 was 1.7 hours. WG3 failed to reach the same final optical density as DSP-10. Similar results were obtained in LB under aerobic conditions (data not shown). However, under anaerobic conditions, no difference in growth rates was observed between WG3 and DSP-10 in the defined medium M1 supplemented with lactate (20 mM) as the electron donor and nitrate (10 mM) or fumarate (10 mM) as the electron acceptor (data not shown). As well, the ability to reduce soluble Fe(III) or Co(II) was not affected in WG3 in anaerobic M1 medium using lactate (20 mM) as the electron donor. This infers that the function of SO1377 is not involved in dissimilatory reduction of metals.

### Transcriptional profile differentiation between mutant and wild type strain

Whole genome microarrays were used to identify genes whose transcription was affected by the deletion of *SO1377 *gene. Gene expression in the mutant WG3 cells, recovered from log phase growth in aerobic MR2A medium, was compared with the expression of DSP-10 cells using the same growth conditions. Statistically (P < 0.05 as cut-off value in *t*-test), transcription of 9.9% of the genes (total 453) was affected by the deletion of *SO1377 *gene (Table 2). The magnitude of expression alteration of these genes was up to 30-fold. Of 453 genes, 42, 42 and 33 were involved in 'energy metabolism', 'cellular processes' and 'transport and binding proteins', respectively (Table 1S, online supplemental material). Significant changes of transcription occurred in 153 hypothetical proteins. As expected, the transcription of *SO1377 *gene was not detected in the mutant WG3 cells but highly expressed in parental strain DSP-10. Meanwhile, *SO1376 *gene, which was predicted to be co-transcribed with *SO1377*, did not have a significant change in transcription relative to the reference control.

The microarray data showed that the mutation of *SO1377 *gene resulted in decreased transcription of genes encoding proteins of siderophore biosynthesis (SO3030, SO3031 and SO3032) and, the outer membrane siderophore receptor (SO3033) (Table 3). Other genes involved in iron transport, binding and storage also exhibited alteration in transcription relative to the wild-type strain (Table 3 and Table 1S). For instance, the *SO3034 *gene, encoding a putative ferric iron reductase, was down-regulated over 2-fold in transcription (Table 3).

The transcriptional profiling of the mutant also revealed that genes involved in oxidative damage protection were up-regulated compared to the wild-type strain (Table 1S). The most significant was SO4405 (KatG-2), a putative heme-containing enzyme that catalyses the dismutation of hydrogen peroxide into water and oxygen, was highly up-regulated in the mutant WG3 and exhibited more than a 5-fold difference in transcription (Table 3). This suggested that the increase of internal oxidative stress might be due to the deletion of *SO1377*.

Note that deletion of the *SO1377 *gene resulted in transcriptional alternation of genes encoding subunits of respiratory electron carriers (Table 1S). Most significantly, the transcription of a cluster of genes which encode subunits of cbb3-type cytochrome c oxidase, such as SO2361 (CcoP), SO2362 (CcoQ), SO2363 (CcoO) and SO2364 (CcoN), was suppressed in the mutant (Table 3). Suppression of complex IV of the respiration chain suggested inhibition of aerobic respiration in the mutant. Consistent with this, the transcriptional level of a cluster of genes encoding SO3134 through 3136, involved in C4-dicarboxylate transport, was down-regulated (Table 3). Unlike ABC-type transporters, which couple ATP hydrolysis to translocation of solute across the cytoplasmic membrane, C4-dicarboxylate transport system belongs to another group of transporters, designated tripartite ATP-independent periplasmic (TRAP) transporters. The driving force of TRAP for solute accumulation comes from an electrochemical ion gradient, which is generated from aerobic respiration, rather than ATP hydrolysis [19]. Interestingly, some genes related to anaerobic respiration were up-regulated in the mutant in relation to the wild-type control. For instance, *SO3980 *gene, which encodes a cytochrome c552 nitrite reductase, was up-regulated over 2-fold in the mutant transcription. Also, a cluster of genes encoding subunits of fumarate reductase, including SO0397 (FdrC), SO0398 (FrdA) and SO0399 (FrdB), were up-regulated 3.4, 3.4 and 1.9-fold, respectively (Table 3). This result suggested that the condition used for culturing the bacteria was not strictly aerobic. Instead, it was microaerobic. Under such conditions, genes that were supposed to be expressed only under anaerobic conditions could be induced. Furthermore, our microarray data showed that, under the same conditions, these anaerobically expressed genes were induced at a higher magnitude in the deletion mutant than that of wild-type cells. This may be one kind of compensations to the energy loss in aerobic respiration in mutant cells.

### Proteomic profile differentiation between mutant and wild type strain

**Figure 3:**
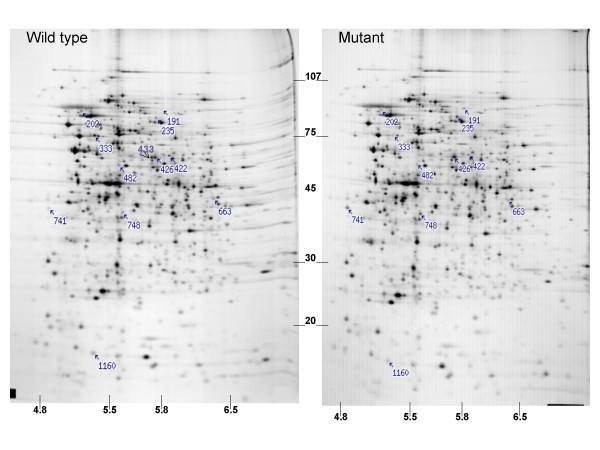
Protein profile differentiation between the *SO1377 *wild-type strain DSP-10 and deletion mutant WG3. Numbers indicated proteins showing statistically significant (P < 0.001) difference in abundance; Note that protein #433 and #333 are absent from the mutant protein pattern. Approximate MW in KDa × 10E-10 is shown on the Y-axis; approximate isoelectric point is shown on the X-axis.

To investigate alterations in protein expression as a result of the *SO1377 *gene mutation, both mutant WG3 and its parental strain DSP-10 cells were subjected to proteomic profile analysis using two-dimensional (2-D) polyacrylamide gel electrophoresis (PAGE) followed by micro liquid chromatography-electrospray ionization tandem mass spectrometry (micro-LC-ESI-MS/MS). Representative 2-D gels of the two strains are presented in Figure 3. Four polypeptides (spot 235, 426, 741 and 748) increased significantly (P < 0.001) in the mutant cells relative to the control. In addition, five polypeptides (spot 202, 422, 482, 553 and 1160) displayed decreased expression and two polypeptides (spot 333 and 433) were not detected at all in WG3 cells (Table 4). Micro-LC-ESI-MS/MS was used to identify proteins showing significant differences in amount on 2-D gels (Table 4). Proteins (spot 333 and 433) completely absent in the deletion mutant were identified as SO1377 (conserved hypothetical protein) and SO2767 (AsnB-1, glutamine-hydrolyzing asparagine synthetase B), respectively. Therefore, similar to microarray analysis, the protein profile verified the deletion of the *SO1377 *gene. Among other identified proteins, those exhibiting lower gel abundance in the mutant, included SO1637 (bacterial surface antigen), SO1117 (putative cytosol aminopeptidase), SO4749 (AtpA, alpha subunit of ATP synthase F1), and SO3466 (RibH, beta subunit of riboflavin synthase). Note that consistent with microarray data, the lower gel abundance of SO4749, the alpha subunit of ATP synthase F1, suggested inhibition of aerobic respiration. The increased gel abundance of proteins included SO2407 (conserved hypothetical protein) and SO3505 (NagA). Again, the increased gel abundance of SO3505 agreed with the microarray data, in which the transcription of SO3505 gene displayed an increase at the magnitude of 2.72-fold in the mutant cells (Table 3). However, the remaining proteins shown in the proteomics data did not exhibit transcriptional changes. One possible explanation for this inconsistence between microarray and proteomics data is that the expression of these proteins is subjected to post-transcriptional regulation.

### Phenotypic differences between the mutant and wild type strain

**Figure 4:**
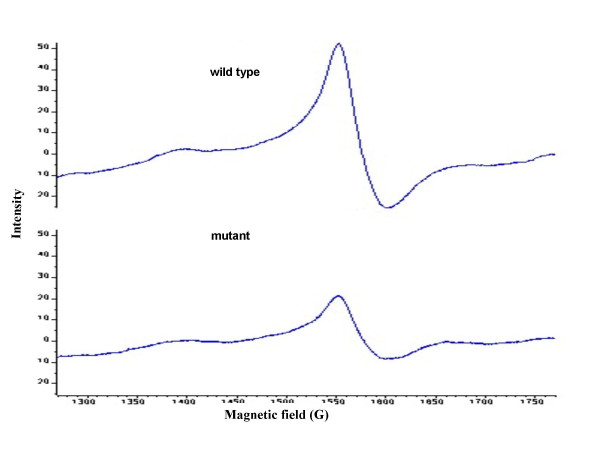
Iron EPR signal from whole-cell preparations. Both *SO1377 *wild-type strain (DSP-10) and mutant (WG3) were cultured in LB liquid for 24 h at 30°C, and EPR samples were prepared as described under "methods". Cell density was adjusted to equal before filling in the quartz EPR tubes according to measured OD_600 nm _Value of each strain. The major signal appears at g = 4.3.

**Figure 5:**
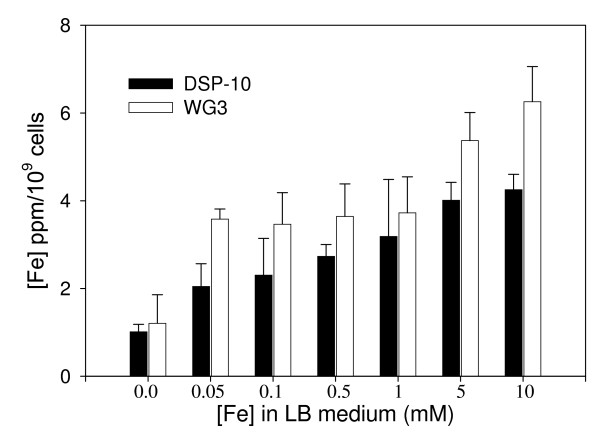
Intracellular iron concentration in both *SO1377 *wild-type strain DSP-10 and mutant WG3 after overnight growth in LB medium supplemented with iron (as ferric citrate). The implemented iron concentration in the medium is shown on the X-axis while the determined intracellular iron is shown in the Y-axis.

**Figure 6:**
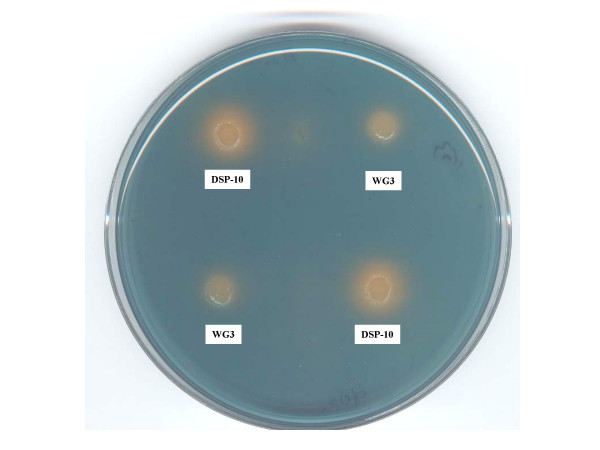
Siderophore production comparison between the *SO1377 *wild-type strain DSP-10 and mutant WG3 on a CAS siderophore testing agar. The chelator-iron (III) complex tints the agar with a rich blue background. The orange halo surrounding the colony indicates the excretion of siderophore and its dimension approximates the amount of siderphore excreted.

As shown in Figure 4, 5 and 6, iron metabolism was altered in the mutant WG3 when compared with the parental strain DSP-10. Whole cell low temperature EPR assay showed that WG3 accumulated 3 times less 'free' iron than DSP-10 (Figure 4), although its total cellular iron level remained largely unchanged (Figure 5). When WG3 and DSP-10 cells were grown on iron-depleted CAS medium, WG3 had decreased siderophore production (Figure 6).

**Figure 7:**
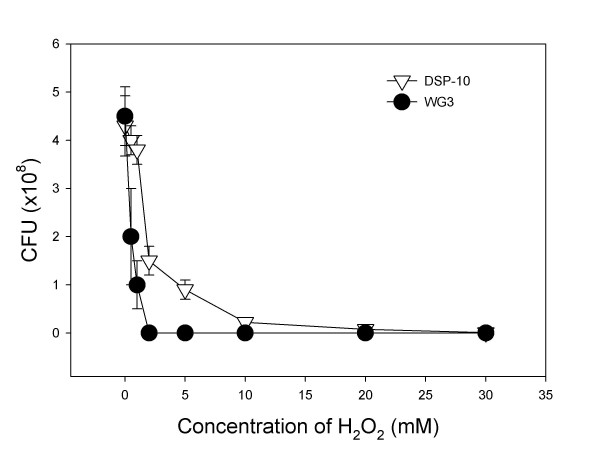
Survival curve of the *SO1377 *wild-type strain DSP-10 and mutant WG3 after subjected to H_2_O_2 _treatment. Note that almost 100% mutant cells cannot survive after 2 mM H_2_O_2 _treatment while about 30 % wild-type cells can survive under the same conditions.

As shown in Figure 7, mutant WG3 was more sensitive to hydrogen peroxide treatment, compared to the parental strain DSP-10. Almost 100% of the mutant cells were killed by 2 mM hydrogen peroxide treatment whereas over 30% wild type cells survived. To kill all wild type cells, 10 mM hydrogen peroxide was needed. This indicated that the mutant became more sensitive to hydrogen peroxide challenge.

Mutant WG3 generated spontaneous mutations at a much higher rate than its parental strain DSP-10. In this study, both gentamicin and kanamycin resistance were used as markers to measure the spontaneous mutation rate. After two days incubation, an average of 144 gentamycin resistant CFU (Colony Forming Unit) arose from WG3, whereas none appeared from DSP-10. The average CFU for WG3 and DSP-10 was 3.27 × 10^9^/ml and 1.18 × 10^10^/ml, respectively. Therefore, it was estimated that the spontaneous mutation rate of gentamicin resistance from WG3 was 4.4 × 10^-7 ^whereas the control was less than 10^-10^. Similarly, the estimated spontaneous mutation rate of kanamycin resistance of WG3 was 3.6 × 10^-6 ^whereas the control was 3.4 × 10^-10^, respectively.

## Discussion

Previously, a 2-D gel expression study revealed that the expression of protein SO1377 was down-regulated during aerobic respiration in a *S. oneidensis *FUR (ferric uptake regulator) mutant [11]. We were curious if the protein was involved, directly or indirectly, in iron metabolism of *S. oneidensis *MR-1. Results from this study provided evidence for this hypothesis. The deletion of *SO1377 *gene altered iron metabolism. Compared with the wild-type control, the mutant accumulated 3 times less intracellular 'free' iron, although the change of total intracellular iron was not evident. Siderophore secretion was lower in the mutant. This was supported by the microarray analysis which showed that the expressions of some genes involved in iron metabolism were altered.

Our study showed that the disruption of *SO1377 *affected other aspects of cellular activities of *S. oneidensis*. *SO1377 *mutant WG3 was highly sensitive to hydrogen peroxide challenge and had a higher spontaneous, mutation rate of other genes. Microarray analysis showed that even without H_2_O_2 _challenge, some genes involved in oxidative damage protection were up-regulated, presumably in response to an increased level of internal H_2_O_2_, due to deletion of the *SO1377 *gene. These phenotypic changes were typical and well documented for mutants of genes involved in iron metabolism and/or oxidative damage protection [20-22]. For instance, the study carried out by Touati *et al *(1995) showed that the permanent derepression of iron assimilation systems in *a fur *deletion mutant of *E. coli *produced an oxidative stress and DNA damage including lethal and mutagenic lesions [23].

Microarray/proteomic analysis also suggests that dysfunction of *SO1377 *affects the proteins involved in respiratory electron transfer and thus respiration functions. The overall trend shows, genes involved in aerobic respiration down-regulate, whereas those involved in anaerobic respiration up-regulate. This coincides with the phenotype observation showing that the mutant has a growth deficiency under aerobic but not anaerobic conditions. This suggests that SO1377 perhaps plays a role in oxidative damage protection in the wild-type strain. Without protection from SO1377, cells tend to down-regulate their aerobic respiration and are more prone to produce free radicals under aerobic conditions. Probably as compensation, bacterial anaerobic respiration pathways were up-regulated in the mutant cells. With the same reasoning, decreased secretion of siderophore, for iron absorption and less accumulation of loose intracellular iron in the mutant should be viewed as adaptation responses of bacterial cells to the loss of SO1377 function. Nevertheless, without SO1377, the bacterium becomes more susceptible to H_2_O_2 _treatment and gives rise to spontaneous mutation of other genes at a level much higher than that of the wild-type strain.

Little is known about the action of SO1377 and how it biochemically interacts with other proteins is still a speculative issue. Since no sequence/structure clues suggest that SO1377 could directly interact with iron or any other metals, it is more likely that the phenotypes derived from disruption of *SO1377 *is indirect. That is, through interactions with other proteins, SO1377 may indirectly participate in iron metabolism of *S. oneidensis *MR-1.

We envision that SO1377 is likely to function as an accessory protein participating in intracellular iron trafficking in *S. oneidensis *MR-1. Evidence supporting this idea comes from several lines. The N-terminal sequence of SO1377 belongs to the PHB protein family. Computational analysis suggests that SO1377 (referred as 4840) has potential functions in formation of protein complexes at the inner bacterial cell membrane, ATP/GTP binding, nucleotide binding, protein transport and, molecular chaperone [18]. TC-BLAST searching shows that partial sequences of SO1376 have similarities with ATX2 and BSD2 of *S. cerevisiae*, respectively . In *S. cerevisiae*, ATX2 encodes a manganese-trafficking protein that localizes to Golgi-like vesicles [24], whereas BSD2 encodes a copper homeostasis factor [25]. Also, SO1376 has four cysteine residues at positions 19, 68, 121 and 136, as well as two histidine residues at positions 151 and 155, suggesting that this protein, of 212 amino acids, has potential to be a metalloprotein. Still, a GES hydrophopathy analysis infers SO1376 is a membrane protein. These clues suggest that SO1376 could play a role as a metallochaperone, directly participating in intracellular iron trafficking. Working together, a presumed SO1376/1377 complex could receive ferrous iron from an upstream protein, i.e. SO3034 (a putative ferric iron reductase protein whose transcriptional level was down-regulated 2.2-fold in the *SO1377 *mutant cells, see Table 3), and then transfer it to downstream proteins, such as those involved in cytochrome heme biosynthesis or Fe-S center biosynthesis, etc. The dysfunction of SO1377 could impair the function of SO1376 and compromise the effective process of intracellular iron (II) trafficking resulting in the free iron 'leaking' into the cytoplasm of cells. As a highly reactive species, the 'leaking' free iron could generate very toxic radicals under aerobic conditions, resulting in hypersensitivity to oxidative stress, presumably as a result of Fenton chemistry. Consequently, this elicits DNA damage and onset of SOS response. This may explain why the *SO1377 *mutant has a higher level of gentamicin/kanamycin resistant mutants.

## Conclusion

We continued our efforts to elucidate the functions of SO1377, a hypothetical protein with a homolog domain to prohibitin of eukaryotes, by generating an in-frame deletion mutant of this gene, followed by a series of experiments to characterize its phenotypic and physiological changes compared with the wild-type strain. Our studies showed that the mutant had a decreased growth rate when cultured aerobically and not affected under anaerobic conditions. Whole-genome expression (RNA and protein) profiles revealed numerous gene and protein expression changes relative to the wild-type strain, including some involved in iron metabolism, oxidative damage protection and respiratory electron transfer, e. g. complex IV of the respiration chain. Although total intracellular iron levels remained unchanged, whole-cell EPR demonstrated that the level of loose iron in mutant cells was 3 times less than wild type cells. Siderophore excretion in the mutant also decreased in iron-depleted medium. Compared with the wild type strain, the mutant was more sensitive to hydrogen peroxide and gave rise to 100 times more colonies resistant to gentamicin or kanamycin. Our study showed that deletion of *SO1377 *gene had pleiotropic effects suggesting that SO1377 perhaps is involved in the iron metabolism and oxidative damage protection of *S. oneidensis *MR-1.

## Methods

### Bacterial strains and plasmid

Bacterial strains and plasmids used in this study are listed in Table 1.

### Culture media and growth conditions

*E. coli *strains were grown at 37°C in LB medium overnight [29]. *S. oneidensis *strains were grown at 30°C in either LB or modified R2A (MR2A) medium [30]. Antibiotics were used at the following final concentrations: 15-μg/ml gentamicin (Gm), 50-μg/ml kanamycin (Kan) or 10-μg/ml rifamycin (Rif). For anaerobic growth of *S. oneidensis *MR-1 and its mutants, the defined medium M1 [per liter: 1.19-g (NH_4_)_2_SO_4_, 0.56-g KH_2_PO_4_, 0.1 ml (0.115 M) Na_2_SeO_4 _and 10 ml 10X mineral solution) was used as described previously [30]. The electron donor in M1 medium was lactate (20 mM, pH7.0), and electron acceptors were ferric citrate (10 mM), KNO_3 _(10 mM) or fumarate (10 mM).

### In-frame deletion of *SO1377 *gene

In-frame deletion of the *SO1377 *gene was generated by the method of Link *et al *[31] and its schematic presentation is shown in Figure 1. The deletion process was carried out as follows. In the first step (Fig. 1A), PCR primers were used to amplify 5'- and 3'- end fragments of *SO1377 *gene, respectively. Primers *SO1377*-No (5'-CGCGAGCTCGCCTTAGCCCTCCTCATCGG-3') and Ni (5'-tgtttaaacttagtggatgggTCCTTTCGTTTACCTACACA-3') were for the 5'-end fragment, and *SO1377*-Ci (5'-cccatccactaagtttaaacaTTGAGCCAAAGATAAGTAAT-3') and Co (5'-CGCGAGCTCGCTCCATGGCAAATAGCCGC-3') for the 3'-end fragment. The outside primers (No and Co) harbor a *Sac*I restriction site. The inside primers (Ni and Ci) contain complementary 21-nt tags at their respective 5' termini as shown in lower case letters. The 21-nt complementary tags used in this study also contain a *Pme*I restriction site. Amplification was performed in a 20-μl volume containing 0.2-μl *Taq *polymerase (Sigma), 2-μl 10X PCR buffer (Promega), 1-μl each primer (20 pmol/μl), 1.2-μl 25 mM MgCl_2_, 1-μl genomic DNA of *S. oneidensis *MR-1 as template (50 ng/μl), 0.5-μl 10 mM dNTP Mix (Clontech) and 13.1-μl ddH_2_O. The PCR mixture was denatured at 94°C for 2 minutes, followed by 30 cycles of 94°C for 30 seconds, 58°C for 1 minute and 72°C for 1 minute, followed by 7 minutes at 72°C. The PCR products were then purified from agarose gel using QIAquick Gel Purification Kit (QIAGEN Inc.). In the second step (Fig. 1A), the 5'- and 3'- end fragments were annealed at their overlapping region and amplified by PCR as a single fragment, using the outer primers (*SO1377*-No and *SO1377*-Co). The PCR amplification conditions were as described above except that the genomic DNA template was replaced by adding 0.5-μl each of 5'- and 3'- end fragments of *SO1377 *amplified in the first round PCR. The fusion product was purified from agarose gel using QIAquick Gel Purification Kit (QIAGEN Inc.), resuspended in 50-μl of 1X *Sac*I restriction buffer containing 5 U of *Sac*I restriction enzyme, and digested for two hours at 37°C. The digested fragment was gel purified, ligated into *Sac*I-digested and phosphatase-treated suicide vector pDS3.0, electroporated into *E. coli *S17-1/λ_pir_, spread on LB gentamicin agar plates and incubated at 37°C overnight. The transformants were screened for inserts by PCR with *SO1377*-No and Co as primers. In the third step, the suicide plasmid construct was integrated into *S. oneidensis *DSP-10 chromosome by homologous recombination (Fig. 1B). This process was facilitated by conjugal transfer between *E. coli *S17-1/λ_pir _cells harboring the suicide plasmid construct (donor) with strain DSP-10, a spontaneous rifampicin-resistant (Rif^r^) derivative of *S. oneidensis *MR-1 (recipient). *E. coli *transformants and DSP-10 cells were grown separately in LB medium with proper antibiotics overnight, washed in fresh medium, and mixed in a 1:3 ratio (donor: recipient) by spotting onto 0.2 μm Millipore membrane disks. Following an eight-hour incubation at 30°C, the cells were removed from the filter disks, resuspended in medium, and plated onto LB agar supplemented with Gm (15-μg/ml) and Rif (10-μg/ml). Correct integration of the suicide vector was verified by PCR amplification. PCR confirmation of the inserts was accomplished by comparing the sizes of the products amplified from the wild-type and mutant DNA using primers flanking the insertion sites (Fo and Ro) (Fig 1B). The Fo primer sequence is: 5'-TGCAGCCAAAAGCACAGCAC-3'. The Ro primer sequence is: 5'-CGAACTGTTATGCCATAAAT-3'. In the fourth step, to obtain the deletion mutation of *SO1377 *gene, the integrated suicide plasmid had to be resolved from chromosome through recombination (Fig. 1C). This was accomplished by growing the strain carrying the integrated suicide vector in NaCl-less LB liquid overnight, followed by 10, 100 and 1000 dilutions of the culture, and plating 100 μl of each dilution onto LB agar containing 5% sucrose. The plates were then incubated at 30°C for two days. Screen for deletion was accomplished by PCR amplification using the outside Fo and Ro primers. Finally, the deletion was verified through DNA sequencing. The sequencing reaction was performed with ABI Prism Dye Terminator Sequencing Kit (Applied Biosystems, Foster City, CA) and analyzed by ABI 3700 sequencer.

### Microarray transcriptional expression profiling

Microarray fabrication, hybridization, probe labeling, image acquisition and processing were carried out as described previously [11,32,33]. Gene expression analysis was performed using 3 independent microarray experiments, with each slide containing 2 replicate arrays of *S. oneidemis *MR-1 genome [34]. Total cellular RNA, from the *SO1377 *gene deletion mutant WG3 and its parental strain DSP-10 were grown to mid-log-phase in the MR2A medium, then isolated and purified using the TRIzol Reagent (Gibco BRL) according to the manufacturer's instruction. The ratios of mutant samples to the wild-type control were normalized by a trimmed geometric mean [11,32,33]. Standard *t*-test was carried out to determine the significance of gene expression.

### Proteomic profiling using 2-D PAGE analysis of whole-cell lysates

Mid-log-phase cells of WG3 and DSP-10, grown in aerobic MR2A medium, were collected and washed three times with 50 nM Tris-HCL (pH = 7.4) and the cell pellets were frozen until used. Frozen cell pellets were mixed with five volumes of a solution containing 9 M urea, 2% (v/v) 2-mercaptoethanol, 2% (v/v) pH 8–10 ampholytes (Biorad), and 4% (v/v) Nonidet P40. The lysed cells were centrifuged for 10 min at 400,000 g in a Beckman TL100 ultracentrifuge to sediment particulates. The supernatants were analyzed by 2-D PAGE. Isoelectric focusing (IEF) gels were cast as described by Anderson and Anderson [35] using a 2:1 mixture of pH 5–7 and pH 3–10 ampholytes (Biorad). Aliquots of samples containing 40 μg of protein were loaded, in triplicate, onto each gel. After IEF, the gels were equilibrated in a buffer containing sodium dodecyl sulfate (SDS) as described by O'Farrell (1975) [36]. The second-dimension slab gels were cast using a linear gradient of 9–17% PAGE. The equilibrated tube gels were secured to the slab gels using agarose and SDS-PAGE as described previously [35]. The proteins were fixed in the gels by soaking in a solution of 20% ethanol (v/v) with 1% formaldehyde (v/v) and detected by silver stain [37]. The 2-DE images were digitized using an Eikonixl412 scanner interfaced with a VAX 4000-90 workstation, and resulting image files were converted to tif format. Data analysis was done using Progenesis software for 2-DE pattern analysis (Nonlinear USA). Statistical analysis of the relative abundance of each matched protein spot across the data set was done using a two-tailed Student *t*-test as previously described [37]. Proteins showing statistically significant differences in abundance (i.e., P < 0.05) were then identified on the basis of their tryptic peptide masses and amino acid sequences. Proteins to be identified were cut from one to five replicate gels (number of spots required varied with the abundance of individual proteins), reduced at room temperature with tris (2-carboxyethyl) phosphine (Pierce, Rockport, IL), alkylated with iodoacetamide (Sigma), and digested *in situ *with modified trypsin (Promega Corp., Madison, WI) [38]. The resulting peptides were eluted from the gel with 25 mM ammonium bicarbonate and 5% formic acid in 50% acetonitrile and analyzed by micro liquid chromatography-electrospray ionization tandem mass spectrometry (μ-LC-ESI-MS/MS). For μ-LC-ESI-MS/MS, samples were loaded onto a 365 × 100 μm fused silica capillary (FSC) column packed with 5 μm Zorbax XDB-C18 packing material (Agilent Technologies, Palo Alto, CA) at a length of 7–8 cm. The tryptic peptides were separated with a 30-min linear gradient of 0–60% solvent B (80% acetonitrile/0.02% heptafluorobutyric acid), and then entered a LCQ ion-trap mass spectrometer (Thermo Finnigan, San Jose, CA). Tandem mass spectra were automatically collected under computer control during 30-min LC-MS runs. MS/MS spectra were then directly subjected to SEQUEST [39,40] database searches to identify proteins by correlating experimental MS/MS spectra to protein sequences predicted by the *S. oneidensis *MR-1 ORE database available in Genbank.

### Assay of hydrogen peroxide challenge

Assay of hydrogen peroxide challenge was carried out as described previously [23]. Cells grown in LB medium to mid-log-phase stage were distributed into 50 ml Erlenmeyer flasks (5 ml each), and hydrogen peroxide was added at concentrations from 0.1 mM to 10 mM. After 20 min of incubation with shaking at 30°C, treatment was stopped by addition of catalase (Roche Molecular Biochemicals) at 400 U/ml and chilling. Samples were diluted in cold 10^-2 ^MgSO_4 _containing 400 U/ml catalase, followed by plating on LB plates for viable count. Colonies were counted after two days of incubation at 30°C.

### Assay for whole-cell electron paramagnetic resonance (EPR) spectroscopy

The procedure for EPR assay was adapted from the description of Keyer and Imlay [41]. After 24 h growth, with shaking in 50-ml LB liquid at 30°C, the optical density (OD_600 nm_) of the culture was measured. Cells were spun down at room temperature and suspended in fresh 50 ml LB liquid containing 20 mM desferrioxamine (DFO) (Sigma). After incubation with shaking for 20 min at 30°C, cells were spun down, washed with cold 20 mM Tris (pH = 7.4), resuspended in 20 mM Tris (pH = 7.4) containing 10% glycerol to reach the same cell density according to OD_600 nm _value measured, frozen in 3-mm quartz EPR tubes (Wilmad) on dry ice, and stored at -80°C until EPR measurement was performed. The EPR spectra were recorded using Bruker X-band spectrometer equipped with a Varian TE102 and a Varian temperature controller. Samples were maintained at -125°C during the recording of signal.

Parameters used for low temperature Fe(III) EPR were as follows: central field, 1520 G; sweep width, 500 G; resolution, 1024 points; frequency, 9.44 GHz; microwave power, 21.523 mW; receiver gain, 3.17e+4; conversion, 327.680 ms; time constant, 327.680 ms; sweep time, 335.544 s; number of scan, 4. EPR data was processed using Bruker WinEPR program.

### Assay of total iron concentration

Intracellular iron concentration was determined as described previously with some modifications [42]. Inoculated cultures were grown in 20 ml of liquid LB, either with no iron supplement or with an iron supplement of 0.05, 0.1, 0.5, 1, 5 and 10 mM ferric citrate, at 30°C for 12 hours with shaking (120 rpm). The cells were washed four times with fresh cold LB medium (twice with 10 ml and twice with 1 ml), and their OD_600 _was determined. The cell pellet was then dispensed in 10 ml 1 N HNO_3 _and incubated in a waterbath at 95°C for 30 min. After spinning down the cellular debris, at 4000 rpm for 10 min, the supernatants were recovered for determination of iron concentration using a Perkin Elmer Optima 3100XL instrument. Iron concentrations were expressed as ppm/10^9 ^cells.

### Assay of siderophore production

CAS (chrome azurol S) blue agar with iron depletion was used to determine the siderophore secretion of the wild-type and deletion mutant strains [43]. Bacterial cultures grown in LB medium, to mid-log-phase, were collected and washed twice with 0.7% NaCl solution and resuspended in 0.7% NaCl solution. A 5 μl suspension of each strain was spotted on the CAS agar, followed by incubation overnight at 30°C. The excretion of siderophore was indicated by the formation of a yellowish halo area surrounding the colony.

### Spontaneous mutation rate of gentamycin or kanamycin resistance

The measurement of spontaneous mutation rate was performed according to the description of Touati et al (1995) [23]. At the amount of 0.1 ml per plate, bacterial cultures grown in LB liquid to mid-log-phase were spread onto LB agar containing 15 μg/ml gentamicin or 50 μg/ml of kanamycin. A serial dilution of the same liquid cultures were made and spread onto LB agar for counting of colony forming unit (CFU) after incubation of two days at 30°C.

## Authors' contributions

WG conducted most of the experiments, carried out data analysis and drafted the manuscript. YL participated in microarray hybridization and data analysis. LW prepared glass slides for microarray. CSG, SLT and TK conducted proteomics analysis and CSG participated in manuscript writing. DMK constructed the plasmid for gene knock-out. MWF participated in experiment design and manuscript writing. JZ initiated and managed the project and participated in manuscript writing.

## Figures and Tables

**Table 1:** Bacterical strains and plasmids

Strain or plasmid	Relevant characteristics	Source or reference
*E. coli*		
S17-1/λ_pir_	RK2 *tra *regulon, *pir*, host for pir-dependent plasmids	26
WG1	S17-1/λ_pir _strain harboring suicide plasmid pWG3	This study
		
*S. oneidensis*		
MR-1	Wild-type strain	2
DSP-10	Spontaneous rif ampin-resistant derivative of the MR-1, used as the reference strain in replace of MR-1 in this study	11
WG2	Transconjugant between *E. coli *WG1 and *S. oneidensis *DSP-10, containing a chromosomal integration of suicide plasmid pWG3	This study
WG3	In-frame S01377 gene deletion mutant of 1496-bp, derivative from WG2	This study
		
Plasmids		
pCVD422	Suicide plasmid, *bla oriT oriR6K sacB*	27
pBSL142	pBluescript derivative, containing gentamicin -resistance gene	28
pDC3.O	Suicide plasmid generated by cloning the gentamicin-resistance gene from pBSL142 into the EcoRV site of pCV442	This study
pWG3	Suicide plasmid generated by cloning a 1.1 kb DNA fragment containing the mutated S01377 gene with 1496-bp deletion into the Sad site of pDS3.0.	This study

**Table 2:** A Summary of genes performing alerted transcription due to delection of *SO1377 *gene

Function categories^1^	Significant^2^	Total	Ratio (%)
Amino acid biosynthesis	2	76	2.6
Biosynthesis of cofactor, prosthetic groups and carriers	3	104	2.9
Cell envelope	8	170	4.7
Cellular processes	14	238	5.9
Central intermediary metabolism	3	48	6.3
DNA metabolism	3	134	2.2
Energy metabolism	26	276	9.4
Fatty acid and phospholipid metabolism	1	55	1.8
Protein fate	2	180	1.1
Protein synthesis	3	136	2.2
Purines, pyrimidines, nucleosides and nucleotides	1	56	1.8
Regulatory functions	6	200	3.0
Signal transduction	4	63	6.3
Transcription	3	53	5.7
Transport and binding proteins	21	251	8.4
Others/Unknown function	15	553	2.7
Hypothetical proteins	70	2087	3.4

^1^Function categories are based on the annotation of TIGR. ^2^Expression changes are considered as significant (<0.05 probability in Student's t-test and >1.5 cutoff value for fold change).

**Table 3:** Genes performing significant altered transcription in the deletion mutant of *SO1377 *gene

Gene	Symbol	Putative Function	Expression		
			AVG	SE	P-value
Energy metabolism					

*SO0397*	*fdrC*	fumarate reductase cytochrome B subunit	+3.445	0.729	*
*SO0398*	*frdA*	fumarate reductase flavoprotein subunit	+3.409	0.711	*
*SO0399*	*frdB*	fumarate reductase iron-sulfur protein	+1.895	0.336	**

*SO1017*	*nuoF*	NADH dehydrogenase I, F subunit	+9.606	2.178	*
*SO2098*	*hyaB*	quinone-reactive Ni/Fe hydrogenase, large subunit	+3.506	0.937	**

*SO2361*	*ccoP*	cytochrome c oxidase, cbb3-type, subunit III	-2.079	0.036	*****
*SO2362*	*ccoQ*	cytochrome c oxidase, cbb3-type, CcoQ subunit	-6.711	0.099	****
*SO2363*	*ccoO*	cytochrome c oxidase, cbb3-type, subunit II	-27.027	0.046	****
*SO2364*	*ccoN*	cytochrome c oxidase, cbb3-type, subunit I	-7.042	0.033	*****

*SO3034*		ferric iron reductase protein, putative	-2.227	0.040	*****
*SO3980*		cytochrome c552 nitrite reductase	+3.346	0.770	*
*SO4513*		formate dehydrogenase, alpha subunit	+2.408	0.370	***
					
Transport and binding protiens					
*SO0530*		transporter, LysE family	+8.483	1.561	**

*SO3030*	*alcA*	siderophore biosynthesis protein	-2.457	0.038	*****
*SO3031*		siderophore biosynthesis protein, putative	-1.534	0.070	*
*SO3032*		siderophore biosynthesis protein, putative	-2.033	0.038	*****
*SO3033*		ferric alcaligin siderophore receptor	-2.079	0.071	***

*SO3134*	*dctP*	C4-dicarboxylate-binding periplasmic protein	-3.165	0.048	*****
*SO3135*		C4-dicarboxylate transporter, putative	-2.793	0.037	*****
*SO3136*	*dctM*	C4-dicarboxylate transport protein	-1.969	0.051	*****

					
Cellular process					
*SO3065*		colicin V production protein	+3.219	0.580	*
*SO4405*	*katG-2*	catalase/peroxidase HPI	+5.409	1.310	*
					
DNA metabolism					
*SO2081*		ATP-dependent helicase, DinG family	+5.111	1.493	*
					
Cell envelope					
*SO4676*	*kdtA*	3-deoxy-D-manno-octulosonic-acid (KDO) transferase	+2.225	0.475	**
					
Biosynthesis ofcofactors, prosthetic groups, and carriers					

*SO4723*		molybdopterin biosynthesis MoeA protein, putative	+1.748	0.183	***
*SO4724*		molvbdenum cofactor biosynthesis protein A	+2.000	0.132	****

					
Amino acid biosynthesis					
*SO4054*	*metF*	5, 10-methylenetetrahydrofolate reductase	+2.061	0.451	*
					
Central intermediary metabolism					
*SO3505*	*nagA*	N-acetylglucosamine-6-phosphate deacetylase	+2.720	0.264	*****
*SO3628*		glycerate dehydrogenase, degenerate	+4.349	1.469	*
					
Signal transduction					
*SO2366*		response regulator	-30.303	0.028	*****
					
Unknown function					
*SO0796*		GGDEF family protein	+7.824	1.795	*
*SO1312*		TolB domain protein	+4.079	1.690	*
*SO2005*		dskA-type zinc finger protein	+3.096	0.503	***
*SO3506*		SIS domain protein	+3.049	0.667	**

*SO4671*	*glpG*	glpG protein	+2.189	0.216	*****
*SO4672*	*g1pE*	glpE protein	+1.574	0.142	**

1. The average (AVG) expression ratio of the mutant sample to the wild-type control was calculated from 12 replicates together with the respective standard error (SE), a standard *t*-test result (*, P < 0.05; **, P < 0.01; ***, P < 0.001; ****, P < 0.0001; *****, P < 0.00001 probability in Student's *t*-test).

2. All genes shown in here have expression change ratio between the mutant and the wild-type more than 2.0-fold, except for several ones if they belong to an operon with a gene performing expression ratio of more than 2.0-fold.

3. Fold increases is marked as (+) and decreases (-). Expression changes of greater than 2.0-fold are shown in bold font.

4. Genes belonging to a same operon are framed in a rectangle.

**Table 4:** Identification of protiens showing signifinant difference in abundance on 2-D PAGE using MIcro-LC-ESI-MS/MS

Spot No.	Gene	Symbol	Putative function	Volume in wild type	Volume in mutant
202	*SO1637*		bacterial surface antigen	21077 (2592)	10347 (2287)
333	*SO1377*		conserved hypothetical protein	49308 (5917)	0
422	*SO1117*		putative cytosol aminopeptidase	21725 (1716)	10017 (771)
426	*SO2407*		conserved hypothetical protein	71070 (11798)	120011 (11641)
433	*SO2767*	*asnB-1*	asparagine synthetase B, glutamine-hydrolyzing	49614 (5917)	0
482	*SO4749*	*atpA*	ATP synthase F1, alpha subunit	25802 (4670)	12153 (4837)
748	*SO3505*	*nagA*	N-acetylglucosamine-6-phosphate deacetylase	22356 (4561)	37349 (2876)
1160	*SO3466*	*ribH*	riboflavin synthase, beta subunit	43043 (9986)	15730 (3665)
235	ND		ND	12369 (2845)	32860 (6604)
663	ND		ND	19704 (3527)	9644 (1948)
741	ND		ND	11690 (2923)	35919 (4382)

Expression changes are considered as signifinant(<0.001 probability in Stident's *t-test*). Number in parenthesis indicate standard deniation. spot volume is a relative measure of integrated density
